# The Potential Impact of Up-Front Drug Sensitivity Testing on India’s Epidemic of Multi-Drug Resistant Tuberculosis

**DOI:** 10.1371/journal.pone.0131438

**Published:** 2015-07-01

**Authors:** Kuldeep Singh Sachdeva, Neeraj Raizada, Radhey Shyam Gupta, Sreenivas Achuthan Nair, Claudia Denkinger, Chinnambedu Nainarappan Paramasivan, Shubhangi Kulsange, Rahul Thakur, Puneet Dewan, Catharina Boehme, Nimalan Arinaminpathy

**Affiliations:** 1 Central TB Division, Government of India, New Delhi, India; 2 Foundation for Innovative New Diagnostics, New Delhi, India; 3 World Health Organization, India Country Office, New Delhi, India; 4 Bill and Melinda Gates Foundation, New Delhi, India; 5 Department of Infectious Disease Epidemiology, School of Public Health, Imperial College London, London, United Kingdom; University College London, UNITED KINGDOM

## Abstract

**Background:**

In India as elsewhere, multi-drug resistance (MDR) poses a serious challenge in the control of tuberculosis (TB). The End TB strategy, recently approved by the world health assembly, aims to reduce TB deaths by 95% and new cases by 90% between 2015 and 2035. A key pillar of this approach is early diagnosis of tuberculosis, including use of higher-sensitivity diagnostic testing and universal rapid drug susceptibility testing (DST). Despite limitations of current laboratory assays, universal access to rapid DST could become more feasible with the advent of new and emerging technologies. Here we use a mathematical model of TB transmission, calibrated to the TB epidemic in India, to explore the potential impact of a major national scale-up of rapid DST. To inform key parameters in a clinical setting, we take GeneXpert as an example of a technology that could enable such scale-up. We draw from a recent multi-centric demonstration study conducted in India that involved upfront Xpert MTB/RIF testing of all TB suspects.

**Results:**

We find that widespread, public-sector deployment of high-sensitivity diagnostic testing and universal DST appropriately linked with treatment could substantially impact MDR-TB in India. Achieving 75% access over 3 years amongst all cases being diagnosed for TB in the public sector alone could avert over 180,000 cases of MDR-TB (95% CI 44187 – 317077 cases) between 2015 and 2025. Sufficiently wide deployment of Xpert could, moreover, turn an increasing MDR epidemic into a diminishing one. Synergistic effects were observed with assumptions of simultaneously improving MDR-TB treatment outcomes. Our results illustrate the potential impact of new and emerging technologies that enable widespread, timely DST, and the important effect that universal rapid DST in the public sector can have on the MDR-TB epidemic in India.

## Introduction

In India, despite impressive progress in the scale-up of DOTS (Directly Observed Treatment Short course) coverage by the public sector, tuberculosis (TB) remains a pressing public health problem [1], owing partly to a vast and unorganized private sector [2–6]. Even when a TB case is diagnosed in the public sector, the timely ascertainment of drug sensitivity presents additional challenges. Most current methods for DST, although highly accurate, are reference laboratory-based, and thus do not lend themselves to widespread use amongst patients at initial diagnosis of TB. As a result, DST is currently prioritized for patients categorized as ‘high-risk’, including those with known HIV co-infection; those with a history of TB treatment; and those not responding to first-line treatment [7]. These technological and resource limitations are amplified by operational challenges. Under routine programmatic conditions, a substantial proportion of persons in risk groups currently eligible for DST are not tested [8–9]. Among those diagnosed as drug-resistant and eligible for a regimen for multi-drug resistant TB, treatment success rates are currently reported at less than 50% [8].

**Fig 1:**
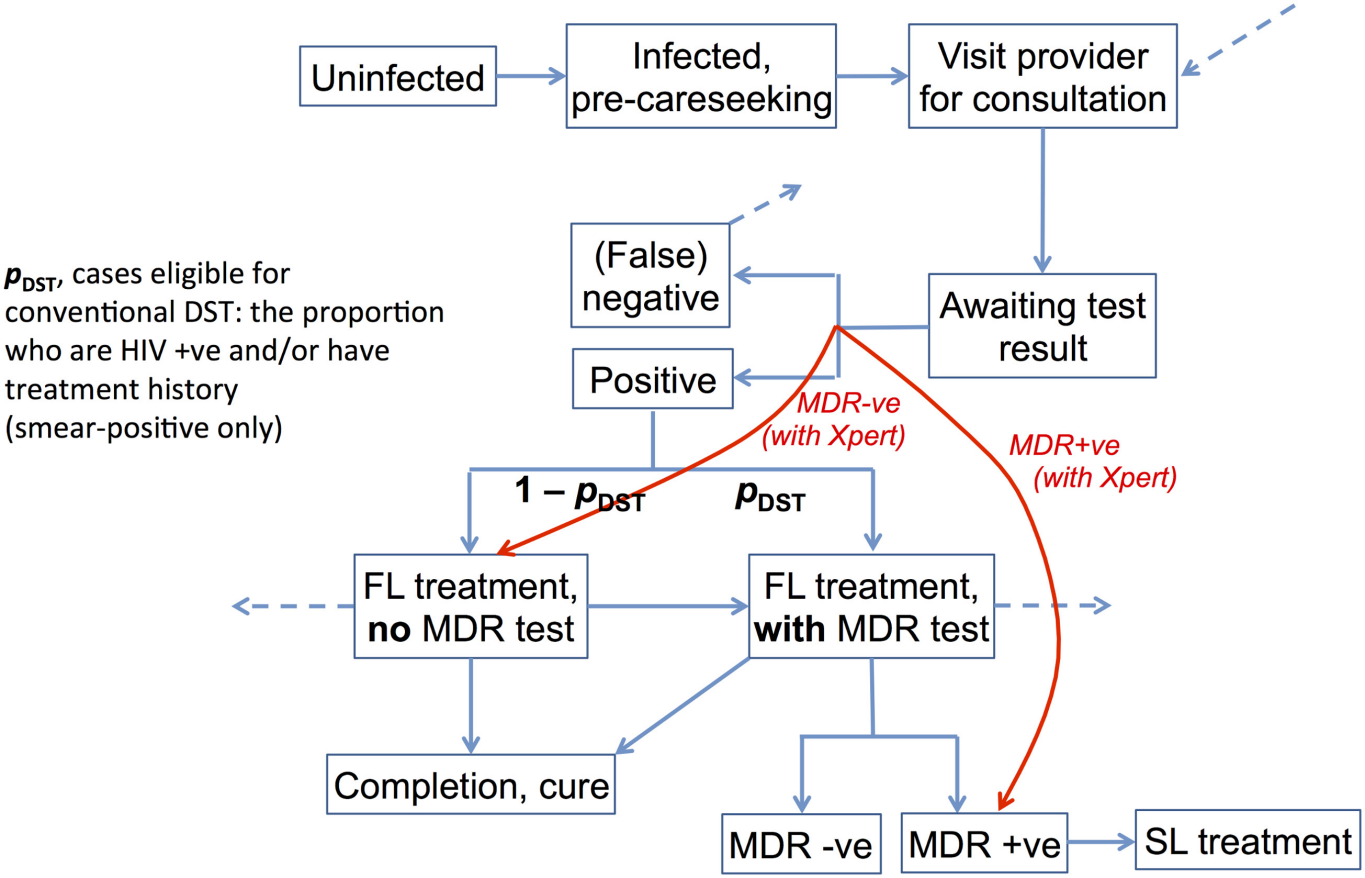
Schematic illustrating the model structure. Infections are stratified by smear, drug sensitivity, HIV co-infection and history of previous treatment. TB care stages (diagnosis and treatment) are stratified by public and private sectors. ‘FL’ and ‘SL’ denote first- and second-line treatment, respectively. Outgoing dashed lines indicate the loss of patients from the care seeking pathway. These patients reenter the care seeking pathway after a given delay, represented by the incoming dashed line at top right. The red lines indicate the effects of Xpert: that is, to bypass the interval for suspecting and testing for MDR-TB, and to enable wider uptake of upfront drug sensitivity testing. Individuals who are cured are assumed to be non-infectious until death, relapse or reinfection. In the latter two cases, they re-enter the infected, pre-care seeking compartment.

The end TB strategy, approved by world health assembly, aims to end the global TB epidemic and targets to reduce TB deaths by 95% and new cases by 90%, between 2015 and 2035. One of the key pillars of this approach is early diagnosis of tuberculosis, including universal drug susceptibility testing, and systematic screening of high-risk groups [10]. New and emerging technologies could make it feasible to meet such conditions, by lifting the constraints associated with current methods of DST, thereby extending high-quality DST to an increased number of patients. A prominent example of such technology is the Xpert MTB/RIF assay [11] (hereafter referred to as ‘Xpert’), a rapid highly-automated nucleic acid amplification test that can provide a highly-accurate result on *M*. *tuberculosis* presence in sputum and rifampicin-resistance status within 2 hours. By decentralizing DST to the district or sub-district level, Xpert may offer the potential for much wider, timelier DST than is feasible with current tools. Moreover, while conventional DST is performed only after TB diagnosis, an assay such as Xpert allows DST to be run concurrently with diagnosis: we refer to this as ‘upfront’ DST. As illustrated in Fig 1, widened access to upfront DST could lead to earlier detection of MDR-TB, particularly amongst smear-negative cases in which it would otherwise be missed. When linked to effective treatment, this improved detection could lead to reduced opportunities for transmission, thus potentially preventing future cases of MDR-TB.

A recent study [12] addressed the potential impact of Xpert in the TB epidemic in India, highlighting the importance of engaging the private sector in controlling TB transmission: in the present study we build on this to further explore the role of Xpert in the public sector for the control of MDR-TB. We ask: what would be the potential impact of upfront DST (as provided by Xpert) in the public sector, on the incidence of MDR-TB over the next ten years? How might this impact vary with increasing efficiency of DST completion among currently-recognized groups at higher risk of MDR-TB, and additionally for patients outside these risk groups? And with a currently estimated treatment success rate of 48% for MDR-TB [8], how would the impact of expanded DST be shaped by accompanying improvements in treatment success? While cost implications are always important in implementation, the present study focuses solely on potential epidemiological impact. Results of this approach can contribute to the basis for subsequent assessment of cost-effectiveness: whether in the context of Xpert, or any other platform for decentralized, rapid DST.

We use a transmission modeling framework to address these questions: such an approach allows us to capture the indirect (transmission-reducing) effects of expanding DST eligibility, earlier MDR diagnosis, and better coverage of diagnostic and treatment services under the RNTCP, as well as providing a systematic framework for bringing together different sources of data on the performance of Xpert relative to current practice. We draw from a multi-centric demonstration project in India conducted under RNTCP, wherein upfront Xpert testing for all presumptive TB & DR-TB cases was piloted under uncontrolled programmatic settings. This study looked into the various programmatic aspects such as feasibility of upfront Xpert testing and the impact of Xpert in a pragmatic, ‘real-world’ scenario [13–16]. In the current manuscript we draw on findings from this [16] study and describe the essential model structure and calibration procedure, with further details given in the appendix (S1 File). We then present results of the model calibrated to the TB and MDR-TB epidemic in India, along with findings for the potential impact of Xpert at different levels of access. We then present sensitivity analysis to key model parameters, relating to the importance of the private sector. Finally we discuss these findings, along with implications for future work.

## Transmission Model

### Model Structure

**Fig 2:**
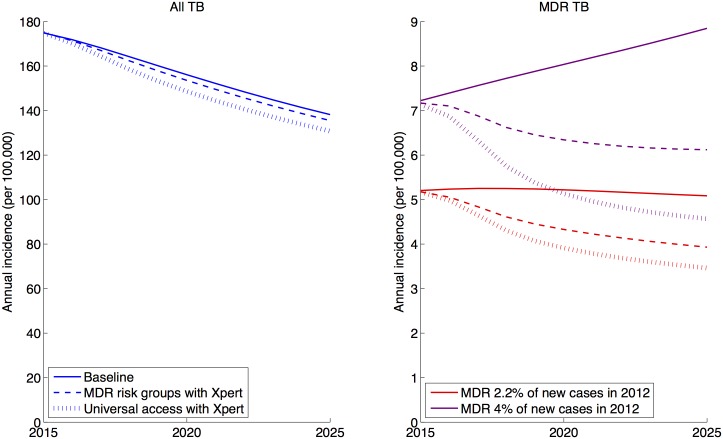
Illustrative results of Xpert scale-up in the public sector on all TB incidence and MDR-TB incidence, assuming 3-year rollout to 100% coverage of the groups shown. Here, ‘baseline’ implies no Xpert, and current levels of DST amongst current MDR risk-groups (smear-positive HIV co-infected and previously-treated cases). Xpert scenarios further include smear-negative cases amongst MDR risk groups. On the right-hand (MDR) panel, red curves correspond to the case where MDR accounts for 2.2% of new cases in 2012, while the purple curves correspond to MDR-TB being 4% of new cases in 2012.

The essential structure of the transmission model is illustrated in Figs 1 and 2, with model parameters given in Table 1 and model equations shown in the appendix (S1 File): here the population is partitioned into different ‘compartments’, reflecting individuals’ state of infection; their care seeking; and their stages of diagnosis, treatment and cure. Rates of transition between these states are captured by a system of ordinary differential equations. The model distinguishes drug-susceptible and MDR-TB, as well as stratifying active disease by smear negative and smear positive status. Here, we regard ‘smear-negative’ TB as being less infectious than smear positive TB, and undetectable by smear microscopy (although potentially still diagnosed clinically—see below).

**Table 1:** Summary of parameters used in the model.

Parameter	Value	Source	Range
***β_rs_*, Transmission rate (mean number of infections per year in a fully susceptible population)**			
* β_01_* Drug-sensitive, sm +	12.2	Fitted	
* β_00_* Drug-sensitive, sm −	0.22 *β_01_*	Multiplicative factor: [32]	[0.1–0.4]
* β_11_* MDR, sm+	5.2	Fitted	
* β_10_* MDR, sm −	0.22 *β_11_*	As above	[0.1–0.4]
***u*_h_, Proportion of new infections having HIV status *h***			
* u*_1_ HIV+ proportion	0.05	[1]	[0.025–0.1]
***p*_h_, Proportion of new infections with HIV status *h* being ‘rapid’ progressors to active disease**			
* p*_1_ (HIV+)	0.37	[33]	[0.25–0.5]
* p*_0_ (HIV−)	0.14	[34]	[0.05–0.25]
***r*_h_, Rate of breakdown to active disease amongst infections with HIV status *h***			
* r*_0_ (HIV+)	0.001	[35]	[0.0005–0.002]
* r*_1_ (HIV−)	0.023	[35]	[0.01–0.04]
***v*_hs_, Proportion of new, active cases with HIV status *h* having smear status *s***			
* v*_00_	0.47	[36]	[0.3–0.6]
* v*_10_	0.56	[36]	[0.45–0.7]
***ρ*, Per-capita relapse rate, all cured TB**			
	0.003	[37]	[0.002–0.004]
***η*, Immunity (reduction in susceptibility) owing to past infection, all cured TB**			
	40%	Assumption	[20–80]
***c*, Rate of initial careseeking (initial patient delay)**			
	1.3 (i.e. 9.2 months delay)	Fitted	
***c'*, Rate of subsequent careseeking (patient delay between careseeking visits)**			
	6 (i.e. 2 months delay)	[17]	[4–12] (i.e. 1–3 months)
**σ_p_, Proportion of patients choosing public/private provider per visit**			
	σ_0_, private-sector preference	0.76	Fitted
***K*_D_, Relative standard of *diagnosis* in private sector, relative to public sector**			
	0.75	Assumption	[0.40, 0.80]
***K*_T_, Relative standard of *treatment* in private sector, relative to public sector**			
	0.5	Assumption	[0.30, 0.70]
***X_ht_*, Proportion of public-sector visits resulting in a test with Xpert**			
	Control parameter (dependent on HIV status *h* and treatment history *t*)		
***d*^(TB)^_sp_, Probability of TB diagnosis (without Xpert)**			
* d* ^(TB)^_11_ (sm+, public)	1	Assumption	
* d* ^(TB)^_10_ (sm+, private)	0.75	*K*_D_ *d* ^(TB)^_11_	Varied by *K*_D_
* d* ^(TB)^_01_ (sm−, public)	0.30	Assumption	[0.15–0.60]
* d* ^(TB)^_00_ (sm−, private)	0.23	*K*_D_ *d* ^(TB)^_01_	Varied by *K*_D_
***d*^(TBX)^_sp_, Probability of TB diagnosis, with Xpert (public sector only)**			
* d* ^(TBX)^_11_ (sm+, public)	1	Assumption	
* d* ^(TBX)^_01_ (sm−, public)	0.45	Fitted to give 11% increase in overall notifications	
***m*, Of MDR-TB cases diagnosed as TB using Xpert, proportion further identified as drug-resistant (public sector only)**			
	0.95	[8]	
***ω*, Rate of treatment initiation**			
	146 (equivalent to 2.5 days	[4]	[365–52]
	treatment delay)		(i.e. 1 day–1 week)
***p*^(DST)^*_hts_*, Proportion of patients receiving conventional DST while on first-line treatment**			
* p* ^(DST)^_111_, HIV+ with previous treatment, public	0.40	[16]	[0.3–0.7]
* p* ^(DST)^_011_, HIV− with previous treatment, public	0.18	[16]	[0.1–0.6]
* p* ^(DST)^_110_, HIV+ with previous treatment, private	0.20	*K*_T_ *p* ^(DST)^_111_	Varied by *K*_T_
* p* ^(DST)^_010_, HIV− with previous treatment, private	0.09	*K*_T_ *p* ^(DST)^_010_	Varied by *K*_T_
***τ*, Per-capita rate of completion of treatment regimen**			
* τ_1_*, First-line	2 (i.e. 6 month duration)	Assumption	
* τ_2_*, Second-line	0.5 (i.e. 24 month duration)	Assumption	
***y*_p_, Proportion successfully treated, first-line**			
* y*_01_ Public	0.88	[10]	
* y*_00_ Private	0.44	*K*_T_ *y*_01_	Varied by *K*_T_
***a_rp_*, Per-capita rate of acquisition of MDR-TB while on first-line treatment**			
* a*_01_, public	0.0068	Fitted	
* a*_00_, private	0.034	5*a*_01_	Multiplicative factor: [2–10]
***y'*_p_, Proportion successfully treated, second-line**			
* y*'_1_ Public sector	0.48	[10]	
* y'*_0_ Private sector	0.24	*K*_T_ *z*_1_	Varied by *K*_T_
***μ*, Per-capita mortality rate, non-TB cases**			
		0.015 (Mean lifespan 66 years)	World bank
***μ_hs_*. Per-capita mortality rate, TB cases**			
*μ*_11_, *μ*_10_ HIV+	0.4	[38]	[0.3–0.5]
*μ*_01_ HIV−, smear +	0.24	[39]	[0.15–0.4]
*μ*_00_ HIV−, smear−	0.096	[39]	[0.05–0.15]

To explore different eligibility criteria for upfront drug sensitivity testing, the model also identifies individuals who have previously received treatment, as well as those co-infected with HIV. With WHO estimates suggesting that 5% of TB cases are co-infected with HIV, the latter is not a major driver of the TB epidemic in India. Rather than separately capturing the dynamics of the HIV epidemic, therefore, the model assumes a given ‘background’ level of HIV, translating to a constant probability of new TB cases being co-infected with HIV.

TB patients in India typically visit a series of providers before being correctly diagnosed, and initiating appropriate treatment [4,17–18]. There is evidence to suggest that TB care in the private sector is of a lower quality than in the public sector [2–3,19]. The widespread use of low-quality diagnostic tests delays the correct diagnosis of TB, while inappropriate treatment reduces the prospects for successful cure, as well as potentially increasing the risk of drug resistance [20–21]. To capture this system we assume a simplified patient pathway, wherein patients seek care in either public or private sectors, each sector with their respective probabilities of accurate diagnosis of TB and MDR-TB. Patients failing to be diagnosed undergo a delay before visiting another provider (represented by the dashed lines in Fig 1). We draw estimates of key parameters for these patient pathways from previous studies [17] and a recent systematic review [4] (Table 1). However, there is little quantitative data on the relative quality of diagnosis and of treatment, comparing public to private sectors. Instead in the model, we assume that the quality of diagnosis—defined as the probability of diagnosis per visit to a provider—in the private sector is K_D_ times that in the public sector. Similarly for treatment outcomes, we assume that the private sector has a success rate that is K_T_ times that in the public sector, where K_D_, K_T_ are both numbers between 0 and 1. We take assumed values of K_D_ = 0.75 and K_T_ = 0.50 (Table 1), and later subject the model findings to a sensitivity analysis with respect to these and other parameters held fixed in the analysis. Where assumptions need to be made about the relative quality of care in the private sector e.g. on diagnosis and cure (Table 1), we subsequently explore the importance of these parameters through a sensitivity analysis.

Diagnosis of drug resistance, by contrast, relies more on laboratory testing than on clinical evaluation. Moreover, the potential value of a test such as Xpert would be primarily to widen access to upfront drug sensitivity testing, as described above, rather than to improve the sensitivity of currently used tests such as line-probe assays. Accordingly, we use estimates of Xpert sensitivity for rifampicin resistance drawn from the literature, and model the effects of widened access to upfront DST using such a test. According to demonstration data [16], a study population receiving essentially universal DST saw a significant increase in notifications of MDR-TB. Consistent with findings in ref [16], we do not assume any change in treatment initiation associated with Xpert deployment.

### Baseline Calibration

Owing to a lack of reliable, long-term time series data, we simulate the model to equilibrium to match the 2012 indicators shown in Table 2, and—to reflect gradually declining incidence—apply a 2% annual decline in transmission for subsequent years (through a 2% annual decline in the fitted transmission parameter, *β*). This approach is likely to be a good approximation for the overall, national TB epidemic as it is only slowly varying in time. With our focus here on MDR-TB, however, we depart from previous approaches [12] to allow for the possibility that MDR-TB may not be at equilibrium. In particular, usage of Rifampicin started in India in the 1980s, with MDR-TB reaching an estimated 3% among new cases [22]. Allowing for this increasing trend over time accommodates a higher fitness for MDR-TB than what might be suggested by an equilibrium scenario. We therefore assume an ‘effective duration’ *D* for the MDR-TB epidemic, that is the number of years prior to 2012 for which it has been in progress (we refer to the ‘effective’ duration since, for simplicity, we do not aim here to model the dynamics associated with DOTS scale-up and changing patterns of care seeking over the last few decades). Starting from the equilibrium solution in the absence of MDR-TB, we initiate the MDR epidemic; simulate forward over *D* years to 2012; and then calibrate the relative fitness of MDR-TB in order to yield the proportions in Table 1, of MDR-TB amongst new and retreatment cases in 2012 as estimated by WHO from sub-national population-based anti-TB drug resistance surveys [23–24]. We assume *D* to be 30 years, consistent with the increasing use of rifampicin in the 1980s [25], incorporating an interval of ten years in either direction.

**Table 2:** Annual incidence and prevalence data used for model calibration: all figures are taken from reference [1].

Indicator	Value	Range
Annual, estimated incidence per 100,000	176	159–193
Estimated prevalence, all forms TB per 100,000	230	155–319
Notified cases, all forms TB per 100,000	119	107–131*
Estimates % of new cases having MDR-TB	2.2	0.8–5.2
Estimated % of previously-treated cases having MDR-TB	15	5.5–38

* Nominal 10% error added for the purpose of the model calibration

Together, incidence and prevalence give an indication of the mean duration of TB disease, before successful treatment, cure or death. However, to allow the model consistently to fit both of these indicators, along with the patient delays estimated in reference [4], we follow reference [26] in additionally allowing for an initial, ‘pre-care seeking’ phase in which patients are infectious, but have not yet contacted the health system for care: the duration of this phase is included as one of the parameters to be estimated.

As in current recommendations, we refer to HIV co-infected and previously-treated cases as MDR ‘risk groups’. We refer to ‘Baseline DST access’ as the proportion of patients currently receiving conventional DST in the absence of Xpert, estimated from demonstration data [16]. For example, this is estimated at 40% for smear-positive, previously-treated cases with HIV co-infection (Table 1). As current guidelines limit DST to smear-positive cases, in practice this represents only a limited proportion of all TB cases that are eligible. Similarly in the simulations, we consider different scenarios for the proportion of patients receiving an upfront Xpert test (whether smear-negative or positive), referring to this proportion as the level of ‘access to Xpert’. We further assume a random allocation amongst patients, so that Xpert replaces conventional DST for patients having access to both. At low levels of Xpert coverage, this is therefore conservative with respect to the approach where Xpert is allocated exclusively to patients not having conventional DST.

Additionally, we assume that 10% of patients undergoing a test are lost to follow up, conservatively assuming this to be unchanged by the use of Xpert. These patients are assumed to remain infectious and to re-enter the care seeking pathway via the incoming dashed line in Fig 1. We calibrate the model to reproduce notifications of TB and of MDR-TB, assuming that all notifications come from the public sector, and that the majority (88%) of cases diagnosed as smear-positive TB in the public sector are notified [8]. Calibrating to notifications has two purposes: (i) It provides additional power in estimating the rate of empirical treatment in the public sector, and (ii) It allows the model to capture the implications of a given increase in the case notification rate, a key finding from the national Xpert demonstration study [16]. In particular this allows us to capture, in a simple way, the important role of empirical treatment in the potential impact of Xpert, as reported elsewhere [27].

The model calibration is performed using Bayesian melding [28–29], which provides a systematic approach for incorporating uncertainty in both model inputs and calibration outputs (e.g. incidence and prevalence), to quantify uncertainty in model estimations of the impact of Xpert. Further details on the model structure and on the calibration procedure are provided in the appendix (S1 File). To explore sensitivity to parameters held fixed in the Bayesian procedure, we perform a univariate sensitivity analysis across the range of values shown for each parameter in Table 1. These parameters relate to the history of tuberculosis infection (such as the rate of breakdown to active disease), as well as the quality of care in the private sector (such as the rates of diagnosis and treatment relative to the public sector). All computations were performed in Matlab, version R2013a.

## Results

The key results of the model are shown in Fig 2, comparing the ‘baseline’ (no Xpert) trajectory with different scenarios for patient access to Xpert. As Fig 2 suggests, although the intervention has a modest effect on incidence of TB (left-hand panel), it has the potential to more substantially impact the transmission of MDR-TB (right-hand panel). As substantial uncertainty exists on the true prevalence of MDR-TB in the country, the right-hand panel illustrates different trajectories of MDR-TB captured by the model, under the baseline scenario (2.2% prevalence of MDR-TB among new cases), as well as the scenario where the proportion of new cases having MDR-TB in 2012 is at the upper bound of current WHO estimates (4%). As described in the methods, assuming an ‘effective duration’ for the MDR-TB epidemic accommodates scenarios where this epidemic may in fact be increasing as of 2012 (4% scenario, right-hand figure).

**Fig 3:**
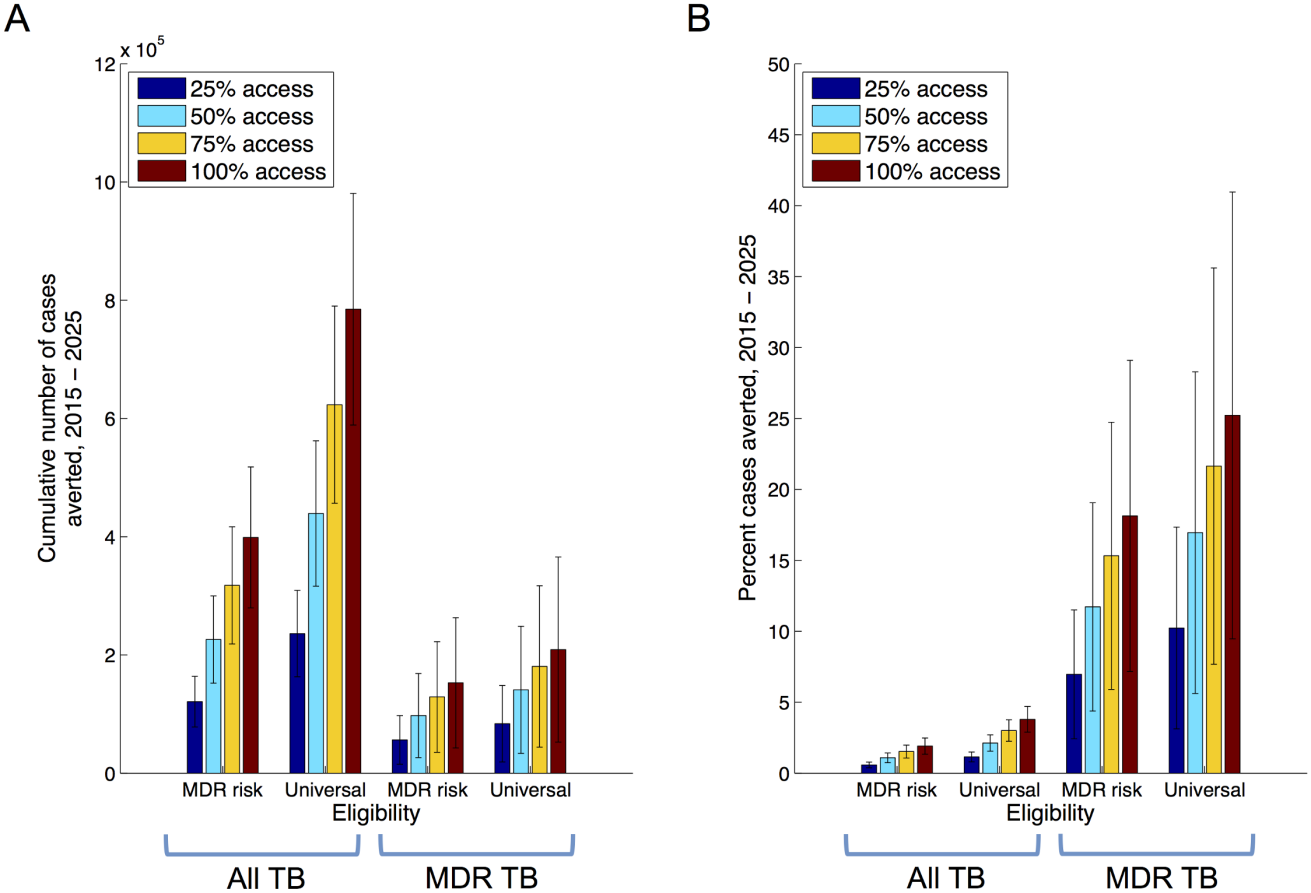
Impact of Xpert deployment in the public sector, at different levels of access. Simulations assume a linear scale-up of Xpert deployment, over a three-year period, to the levels shown here. On the horizontal axis, ‘MDR risk’ denotes those with treatment history and/or HIV co-infection, while ‘Universal’ denotes all TB suspects. Panel A shows the cumulative numbers of cases averted from 2015 to 2025, while panel B expresses this impact as a proportion of the cases that may have occurred under the baseline scenario.

The implications of different levels of access to Xpert in the public sector are shown in Fig 3, with the averted cases between 2015 and 2025 measured as the effect of Xpert on the cumulative incidence over this time. Numerical values supporting this figure are shown in table A in the S1 File. For example, universal eligibility with over 75% access could reduce cumulative MDR incidence by over 20% (Fig 3 -panel B), averting 180,000 cases of MDR-TB (95% CI 44187–317077 cases) between 2015 and 2025. While the relative impact is smaller for All TB cases (Fig 3 -panel B), with the same scenario reducing incidence by 3%, in absolute terms this nonetheless amounts to over 600,000 cases of TB by 2025 (Fig 3 -panel A). Table A in S1 File additionally shows estimates of the number of Xpert tests required under each of the scenarios shown in Fig 3.

**Fig 4:**
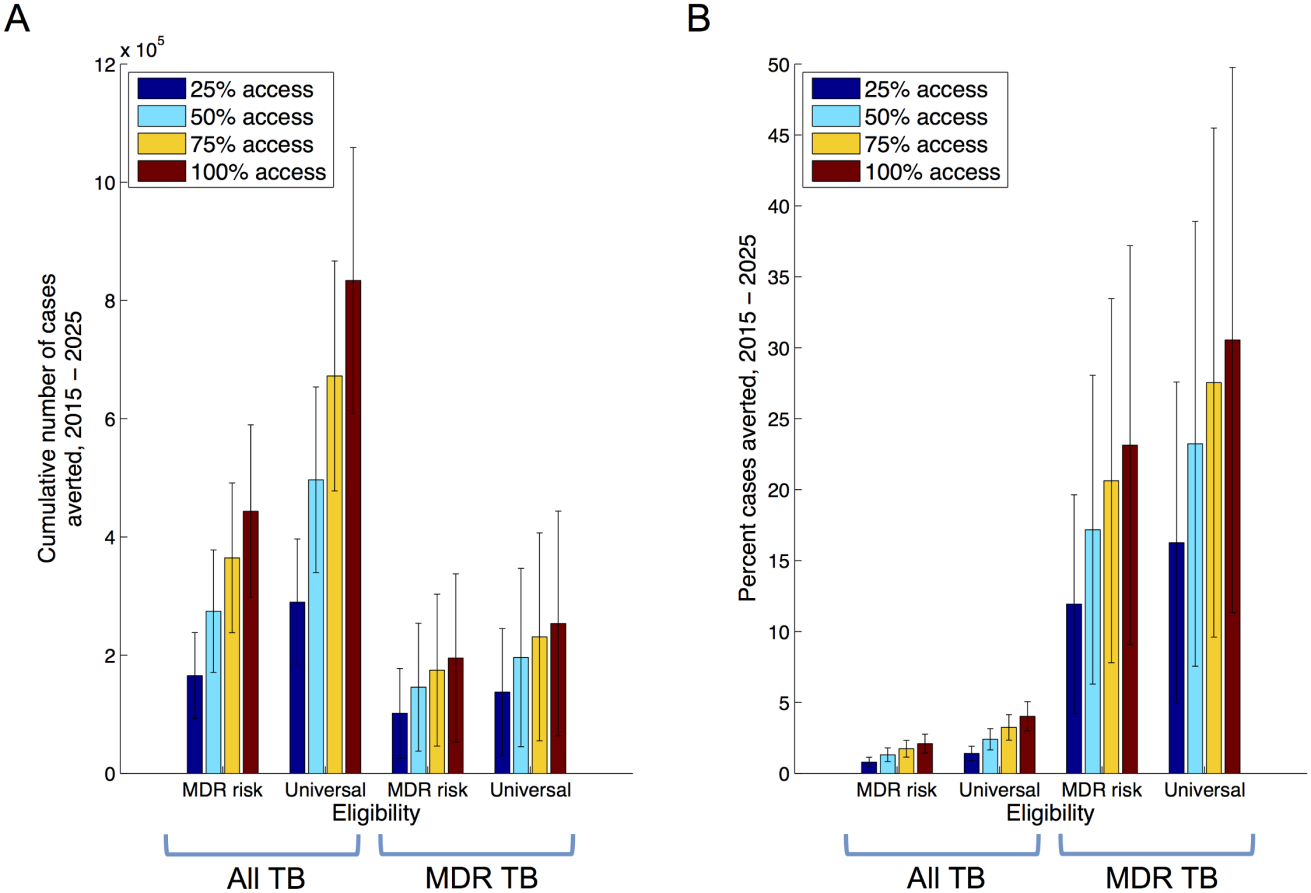
Impact of Xpert deployment when coupled with improved treatment success. Simulations assume a linear scale-up of Xpert deployment (as in Fig 3), along with a linear increase in second-line treatment success in the public sector, over the same three-year period. As in Fig 3, panel A shows the cumulative numbers of cases averted from 2015 to 2025, while panel B expresses this impact as a proportion of the cases that may have occurred under the baseline scenario.

In combination with improved MDR TB treatment, the use of Xpert could reduce incidence still further. In particular, treatment for MDR-TB is costly, protracted and toxic. Currently treatment success in the public sector is roughly 48% [8], a factor that would become increasingly important in a future scenario where increasing numbers of MDR-TB are notified. Accordingly, Fig 4 explores a scenario where second-line treatment success is increased linearly to 85% over three years: although likely an infeasible goal with currently available regimens, this scenario nonetheless demonstrates the synergistic effects that would arise from increased diagnosis coupled with improved cure. This synergy essentially arises from an increased proportion of MDR ascertainment ultimately being converted into cures, rather than remaining infectious.

**Fig 5:**
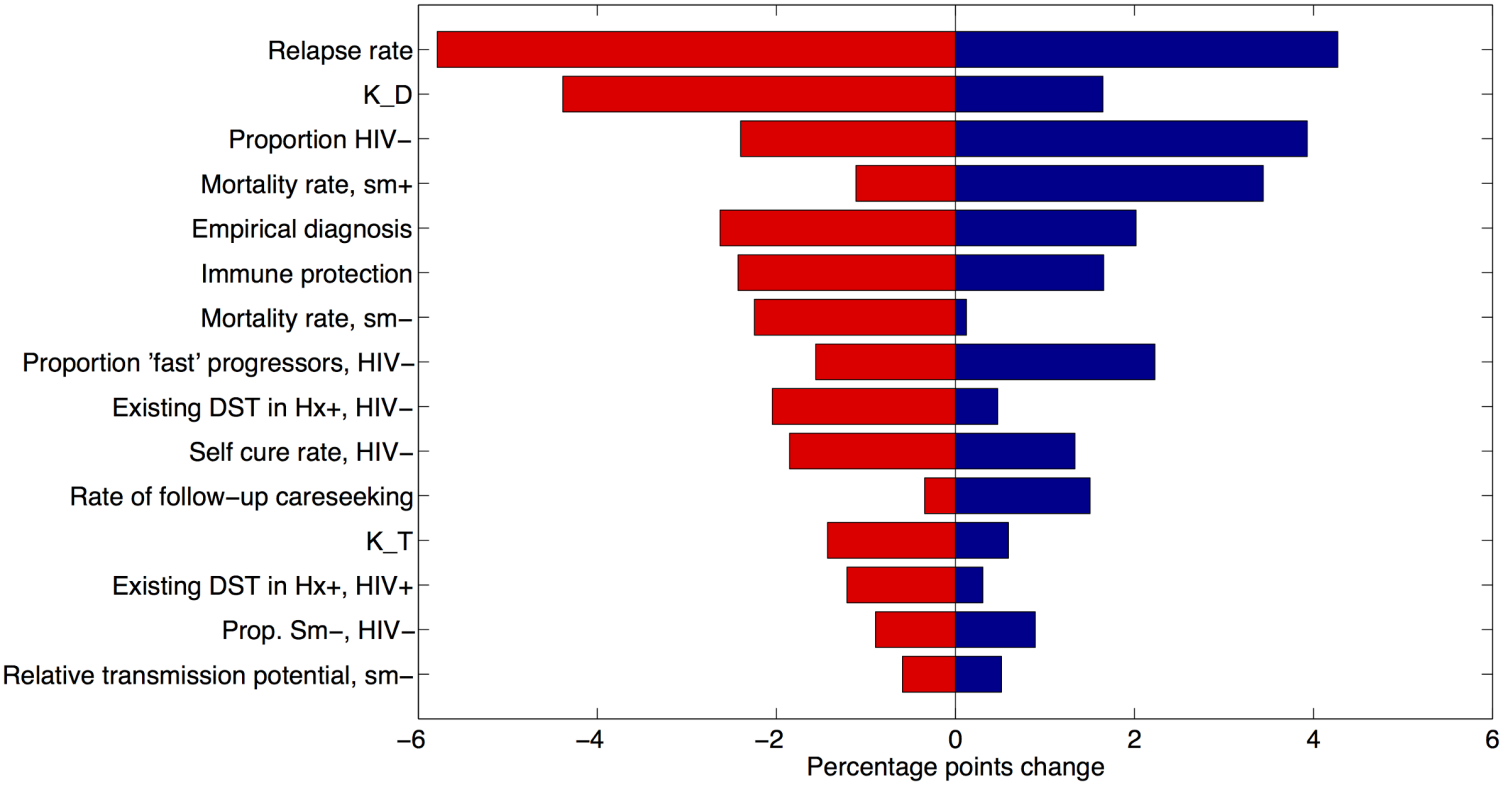
One-way model sensitivity to parameters held fixed in the analysis, conducted with respect to the percent cases averted of MDR-TB at 100% coverage, universal eligibility (rightmost red bar, Fig 3). Shown here are absolute percentage changes, displaying those fifteen parameter ranges to which the model findings are most sensitive. Here, K_D represents the quality of diagnosis in the private sector relative to that in the public sector, defined as the relative probability of diagnosis per visit to a private-sector provider. Similarly, K_T represents the quality of treatment relative to the public sector, measured using the relative probability of cure. See Table 1 for assumed values and ranges for these parameters.

Fig 5 explores one-way sensitivity of the model results to remaining parameters that are held fixed in Figs 3–4. The relapse rate—the most sensitive parameter in these calculations—governs the replenishment of active cases even amongst those who have been cured from active TB disease. Of the remaining, amongst the most important is the relative quality of diagnosis in the private sector (K_D_). Owing to the significance of the private sector in the patient pathway, this parameter plays a key role in the time-to-detection, and hence the infectious period, of a TB patient.

## Discussion

Universal access to high-quality diagnosis and care is a cornerstone of the Stop TB strategy [30]. Today, the complexity and diversity of India’s healthcare system, along with the limitations of current tools, present considerable challenges in reaching this goal. However, new and emerging technologies such as Xpert could offer fresh opportunities in this context. Feasibility and potential impact of such interventions on key programmatic indicators have been reported earlier [13–16]. However, policy recommendations need additionally to take account of the potential long-term impact on TB & DR-TB burden of such an intervention, as well as its cost- effectiveness.

Addressing the first of these needs, the present analysis extends previous work in two important respects: (i) It explores the potential impact of universal rapid DST in the context of an MDR-TB epidemic that may be growing rather than being at equilibrium, and (ii) where possible, it draws realistic parameters arising from a recent, programmatic demonstration of Xpert in the Indian healthcare system. We have explored the potential impact of different levels of access to upfront rapid DST in the public sector, ranging from targeting amongst specific risk groups, and the important influence of service coverage. The model illustrates, for example, that even in the case of an increasing trend in MDR-TB burden, sufficient deployment of Xpert in the public sector could contain this epidemic (Fig 1B). However, improved outcomes from second-line treatment will form an important role in truly controlling MDR-TB (Fig 4), as well as improving the quality of care across the healthcare system to reduce opportunities for the emergence of drug resistance.

More broadly, there are different potential approaches for addressing the MDR-TB epidemic: one is to improve the efficiency of current systems and tools, for example by increasing the proportion of patients receiving a definitive early DST result, and starting appropriate treatment as a result. Important though such efficiencies are, current DST tools (based in central laboratories) would pose substantial challenges in maintaining these efficiencies at scale. Another approach may be to expand the coverage of DST using currently used tests: in principle this would also deliver an impact similar to that shown in Figs 3 and 4, however such approaches could again involve a prohibitive effort given the limitations of currently-used assays for DST. Here, the key benefit offered by an assay such as Xpert is that it potentially allows the scales explored in this study to be practicably achieved, while addressing many of the efficiency concerns by offering a rapid DST result. These efficiency gains could be further enhanced by integrating assays with emerging information and communication technology system such as the Nikshay platform [8], allowing the rapid communication of test results to patients, providers and carers. Moreover, the results of this analysis need not be limited to Xpert: while the analysis draws from recent demonstration data [16] on the effects of Xpert, the essential results apply to any similar diagnostic platform that can facilitate widespread, upfront and rapid DST [31].

While we have concentrated here on epidemiological impact, future work will also address the cost-effectiveness of Xpert deployment in India. In particular, from the policy perspective, it is important for the costs of acquiring and deploying Xpert to be weighed against the costs saved from averted cases of TB. The latter are especially significant in the treatment of MDR-TB, with second-line treatment regimens typically being orders of magnitude more costly than first-line regimens. Despite their small numbers in comparison with drug-sensitive cases, therefore, MDR-TB cases account for a disproportionate amount of TB programme costs in India today [8].

As with any modeling study, our analysis has some limitations to note: first, in calibrating the baseline we assumed that the TB epidemic would be adequately described by parameters derived from an equilibrium model. This would be valid if in reality the ‘true’ dynamics of the TB epidemic are stable, or slowly changing through time. Second, the model outputs depend in large part on the quality of the data used as inputs: we have aimed to address this to some extent using a Bayesian melding algorithm to account for uncertainties in the data, as well as conducting sensitivity analyses to particular parameters. Nonetheless there remain very relevant uncertainties, particularly around the movement of patients through the healthcare system. For example, how long are patients typically infectious before first seeking care? How consistent is public sector utilization across patients? Recent and ongoing studies [4, 17,19], casting increasing light on these questions, will offer more opportunities for understanding the potential role of different interventions in such a complex healthcare system. Finally, the current study focuses exclusively on epidemiological impact, and does not address the cost-effectiveness of the intervention being discussed for the public health programme. In doing so (as noted above), our epidemiological findings could apply more broadly than to GeneXpert, while precise cost implications would depend on the specific diagnostic test being considered (whether GeneXpert or some future rapid diagnostic platform). Forthcoming work addresses this important aspect in the specific context of GeneXpert.

## Conclusion

In conclusion, new and emerging technologies offer novel opportunities for the early diagnosis and treatment of TB. At sufficient scale, and as part of a broader effort to bring closer parity between treatment outcomes for drug-susceptible and drug resistant forms of TB, these interventions could contribute to controlling the transmission of MDR-TB. In doing so, they could play a valuable role in bridging the gap between care and need that plays such a leading role in the TB epidemic in India.

## Supporting Information

S1 File Appendix. The potential impact of upfront drug sensitivity testing for tuberculosis in India.
(PDF)
