# Supplementary material for: The Potential Impact of Up-Front Drug Sensitivity Testing on India’s Epidemic of Multi-Drug Resistant Tuberculosis
Source: PLoS One. 2015 Jul 1;10(7):e0131438. doi: 10.1371/journal.pone.0131438 (PMC4488842; doi:10.1371/journal.pone.0131438)
Supplement: S1 File — The potential impact of upfront drug sensitivity testing for tuberculosis in India. (PDF) [file pone.0131438.s001.pdf]

The potential impact of upfront drug sensitivity testing for tuberculosis in India

Supporting Information

Cases averted and Xpert tests consumed

Table A shows the cases averted in Fig.3, along with the corresponding numbers of tests consumed to acquire these levels of coverage. For the latter, the transmission model only counts cases of TB, but to account for the overall amount of Xpert testing needed, it is necessary also to account for TB-negative suspects receiving Xpert testing. In particular, the demonstration data suggests that roughly 24% of suspects receiving an Xpert test are diagnosed as having TB; amongst MDR risk groups, this figure is 87%.

To estimate the total number of Xpert tests corresponding to a given coverage, we therefore divide the modelled number of TB diagnoses by a factor of 0.24 (under universal access) and a factor of 0.87 (when targeting MDR risk-groups). A simple calculation, this gives indicative numbers for the scale of Xpert deployment involved, and may be an underestimate: a more in-depth analysis would, for example, account more systematically for how these ratios may change over time, with changes in incidence and prevalence.

Table A: Cases averted of TB and MDR-TB from 2015 – 2025, for the simulations shown in Fig.3, as well as the corresponding number of tests consumed (right-most column). All numbers in hundreds of thousands: numbers in parentheses show 95% credible intervals.

Eligibility	Access	All TB	MDR TB	Xpert tests conducted
Baseline, cumulative incidence	-	205.69 (192.10, 221.35)	6.75 (3.44, 12.44)	-
MDR risk groups, averted incidence	25%	1.21 (0.78, 1.64)	0.56 (0.15, 0.98)	33.67 (30.47, 36.86)
	50%	2.26 (1.52, 3.00)	0.98 (0.26, 1.69)	63.50 (58.33, 68.67)
	75%	3.18 (2.19, 4.17)	1.29 (0.35, 2.22)	90.74 (84.15, 97.34)
	100%	3.99 (2.80, 5.18)	1.53 (0.43, 2.63)	115.84 (107.71, 123.98)
Universal access, averted incidence	25%	2.36 (1.63, 3.09)	0.84 (0.19, 1.48)	217.95 (194.39, 241.51)
	50%	4.39 (3.16, 5.62)	1.41 (0.33, 2.49)	417.48 (375.70, 459.26)
	75%	6.23 (4.57, 7.90)	1.81 (0.44, 3.17)	601.79 (545.89, 657.68)
	100%	7.85 (5.89, 9.81)	2.09 (0.53, 3.66)	771.13 (704.72, 837.54)

Further details of the transmission model

1. Model specification

In the following notation it is necessary to label infected states by whether they are infected with drug-sensitive or drug-resistant strains; their treatment history; their smear status, etc. We do this using ordered subscripts, writing X_{htrsp} for a given state X. The subscripts designate the categorisations shown in the following table, and allow a straightforward way of keeping track of the various subdivisions in the model:

Subscript	Value	
h	0: HIV-negative	1: HIV-positive
t	0: Treatment-naive	1: Had previous treatment
r	0: Drug-sensitive TB	1: MDR-TB
s	0: Smear-negative	1: Smear-positive
p	0: Private sector	1: Public sector

Together, for example, the subdivisions h, t designate the 'risk-group' to which a patient belongs, in relation to HIV status and treatment history. Where only some of these subdivisions are relevant to a given state (e.g. smear status not being relevant for latently infected cases), the expressions below only incorporate those relevant subscripts.

In what follows, several terms in the governing equations involve summations over the subscripts. For conciseness we use square brackets to denote these summations so that, for example, $L_{[htr]}$ denotes a summation of L_{htr} over all combinations of the subdivisions h, t and r. Time-derivatives are represented with an overdot (e.g. $\dot{L}_{htr}$).

State variables in the model are organised in terms of the 'stage' of the patient pathway that they relate to. Thus, for example, states $D^{(i)}$ relate to patients in the diagnosis-seeking stage, while states $T^{(i)}$ relate to patients in the treatment stage. Finally, see table 1 in the main text for identification of rates and proportions stated below.

Pre-careseeking stages

Uninfected cases: depletion by infection, and replenishment by population turnover

$$\dot{U} = -U\lambda_{[rs]} + \mu\left[L_{[htr]} + T^{[(i)]}_{[htrsp]} + C^{[(i)]}_{[htrs]}\right] + \sum_{h,s}\mu_{hs}\left[I_{[htrs]} + D^{[(i)]}_{[htrsp]} + B_{[htrsp]}\right] \tag{1}$$

where λ_{htrsp} is the 'force of infection' arising from infectious cases of types h, t, r, s, p, namely:

$$\lambda_{rs} = \beta_{rs}\left[I_{[ht]rs} + D^{[(i)]}_{[ht]rs[p]} + B_{[ht]rs[p]}\right], \tag{2}$$

with infectious terms on the right-hand side defined in the following, remaining equations.

Latent infections without treatment history

$$\dot{L}_{h,0,r} = u_h(1-p_h)\left(U + \eta L_{h,0,[r]} + \eta C^{[(i)]}_{h,0,[r]}\right)\lambda_{r[s]} - (r_h + \mu + \eta p_h \lambda_{r[s]})L_{h,0,r} \tag{3}$$

Latent infections with treatment history

$$\dot{L}_{h,1,r} = u_h(1-p_h)\left(\eta L_{h,1,[r]} + \eta C^{[(i)]}_{h,1,[r]}\right)\lambda_{r[s]} - (r_h + \mu + \eta p_h \lambda_{r[s]})L_{h,1,r} \tag{4}$$

Active disease, pre-careseeking, without treatment history

$$\dot{I}_{h,0,rs} = u_h p_h\left(U + \eta L_{h,0,[r]} + \eta C^{[(i)]}_{h,0,[r]}\right)\lambda_{rs} + r_h v_{hs} L_{h,0,r} - (c + \gamma_h + \mu_{hs})I_{h,0,rs} \tag{5}$$

Active disease, pre-careseeking, with treatment history

$$\dot{I}_{h,1,rs} = u_h p_h\left(\eta L_{h,1,[r]} + \eta C^{[(i)]}_{h,1,[r]}\right)\lambda_{rs} + r_h v_{hs} L_{h,1,r} - (c + \gamma_h + \mu_{hs})I_{h,1,rs} \tag{6}$$

Diagnosis-seeking stages

Patient consulting provider in sector p, awaiting diagnosis without Xpert

$$\dot{D}^{(1)}_{htrsp} = \sigma_p\left(1 - X_{ht}\right)\left(cI_{htrs} + c'B_{htrs}\right) - (g_p + \gamma_h + \mu_{hs})D^{(1)}_{htrsp} \tag{7}$$

Patient consulting provider in sector p, awaiting diagnosis with Xpert

$$\dot{D}^{(2)}_{htrsp} = \sigma_p X_{ht}\left(cI_{htrs} + c'B_{htrs}\right) - (g_p + \gamma_h + \mu_{hs})D^{(2)}_{htrsp} \tag{8}$$

Patient diagnosed as having TB (includes patients with unknown MDR status - also see equ.(17) for patients failing to be diagnosed altogether)

$$\dot{D}^{(3)}_{htrsp} = g_p d^{(TB)}_{sp} D^{(1)}_{htrsp} + g_p d^{(TBX)}_{sp}\left[D^{(2)}_{ht,0,sp} + (1-m)D^{(2)}_{ht,1,sp}\right] - (\omega + \gamma_h + \mu_{hs})D^{(3)}_{htrsp} \tag{9}$$

Patient diagnosed as having MDR-TB - either upfront through Xpert, or subsequently through followup (conventional) DST (applies only to subscript $r = 1$)

$$\dot{D}^{(4)}_{ht,1,sp} = g_p d^{(TBX)}_{sp} m D^{(2)}_{ht,1,sp} + w_1 T^{(2)}_{ht,1,sp} - (\omega + \gamma_h + \mu_{hs})D^{(4)}_{ht,1,sp} \tag{10}$$

Treatment stages

Patient on first-line treatment, without followup DST (laboratory culture, or LPA): negative terms refer respectively to default (δ_p); *de-novo* acquisition of multi-drug-resistance (a_{rp} - assumed zero if the patient already has MDR-TB); followup DST owing to non-response to treatment (f); first-line treatment completion (τ_1); and death (μ_{hs}).

$$\dot{T}^{(1)}_{htrsp} = \omega\left[1 - p^{(DST)}_{hts}\right]\left[D^{(3)}_{htrsp} + D^{(4)}_{htrsp}\right] - (\delta_p + f + a_{rp} + \tau_1 + \mu_{hs})\,T^{(1)}_{htrsp} \tag{11}$$

Patient on first-line treatment, pending results from followup DST (laboratory culture, or LPA): the last term on the right-hand-side denotes cases identified as MDR-TB (entering class $D^{(4)}$, above).

$$\dot{T}^{(2)}_{htrsp} = \omega p^{(DST)}_{hts} \left[D^{(3)}_{htrsp} + D^{(4)}_{htrsp} \right] + fT^{(1)}_{htrsp} - \left(\delta_p + a_{rp} + \tau_1 + \mu_{hs} \right) T^{(2)}_{htrsp} - w_r T^{(2)}_{htrsp} \tag{12}$$

Patient on second-line treatment:

$$\dot{T}^{(3)}_{ht,1,sp} = \omega D^{(4)}_{ht,1,sp} - (\delta_p + \tau_2 + \mu_{hs}) T^{(3)}_{ht,1,sp} \tag{13}$$

Cure and between-careseeking stages

Cure from first-line treatment (noting subscript $t = 1$ on the left-hand-side to denote individuals having treatment history):

$$\dot{C}^{(1)}_{h,1,rs} = \omega y_{rp} \left[T^{(1)}_{htrs[p]} + T^{(2)}_{htrs[p]} \right] - \left(\rho + \eta \lambda_{[rs]} + \mu \right) C^{(1)}_{htrs} \tag{14}$$

Cure from second-line treatment:

$$\dot{C}^{(2)}_{h,1,1,s} = \omega y' T^{(3)}_{ht,1,s[p]} - \left(\rho + \eta \lambda_{[rs]} + \mu \right) C^{(2)}_{ht,1,s} \tag{15}$$

Self-cure

$$\dot{C}^{(3)}_{htr} = \gamma_h \left(I_{ht[rs]} + D^{[(i)]}_{ht[rsp]} \right) - \left(\rho + \eta \lambda_{[rs]} + \mu \right) C^{(3)}_{htr} \tag{16}$$

Between care-seeking episodes: patients failing to be diagnosed, or who have defaulted from treatment:

$$\dot{B}_{htrs} = g_p \left(1 - d^{(TB)}_{sp} \right) D^{(1)}_{htrsp} + g_p \left(1 - d^{(TBX)}_{sp} \right) D^{(2)}_{htrsp} + \delta_p \left(T^{(1)}_{htrsp} + T^{(2)}_{htrsp} \right) + \delta'_p T^{(3)}_{htrsp} - (c' + \mu_{hs}) B_{htrs} \tag{17}$$
